# Scan/rescan reliability of magnetic resonance imaging (MRI)

**DOI:** 10.1007/s00586-025-08649-8

**Published:** 2025-01-19

**Authors:** Menekse Salar Barim, M. Fehmi Capanoglu, Richard F. Sesek, Sean Gallagher, Mark C. Schall, Ronald J. Beyers, Gerard A. Davis

**Affiliations:** 1 Division of Field Studies and Engineering (DFSE), Engineering and Physical Hazards Branch (EPHB), Human Factors and Ergonomics Team (HFET), National Institute of Occupational Safety and Health (NIOSH), 1090 Tusculum Avenue, Cincinnati, OH 45226 USA; 2 Department of Industrial and Systems Engineering, Auburn University, 3301 Shelby Center, Auburn, AL 36849-5346 USA; 3 MRI Research Center, Auburn University, Alabama, USA; 4 Department of Industrial Engineering, Bursa Technical University, Bursa, Türkiye

**Keywords:** Vertebral and intervertebral dimensions, Magnetic resonance imaging, Repeatability, Reliability

## Abstract

**Background:**

Magnetic resonance imaging (MRI) is increasingly used to estimate the geometric dimensions of lower lumbar vertebrae. While MRI-based measurements have demonstrated good reliability with interclass correlation coefficients (ICCs) of 0.80 or higher, many evaluations focus solely on the comparison of identical MRI images. This approach primarily reflects analyst dexterity and does not assess the reliability of the entire process, including imaging and image selection.

**Objective:**

To evaluate the inter- and intra-rater reliability of the entire process of using MRI to measure biomechanically relevant lumbar spinal characteristics, incorporating imaging, image selection, and analysis. Methods: A dataset of 144 low-back MRI scans was analyzed. Reliability assessments were performed under different conditions: (1) identical scans rated by the same analyst at different times (intra-rater reliability) and (2) distinct scans of the same subject obtained by different MRI operators and analyzed by different analysts (inter-rater reliability). Mean absolute differences in measurements were calculated, and sources of variability, such as breathing artifacts, were noted.

**Results:**

Larger discrepancies were observed when comparing distinct scans analyzed by different MRI operators and analysts. In the “worst-case” scenario, where both the MRI operator and analyst differed, a 4.05% mean absolute difference was noted for anterior endplate measurements. This was higher than the 2.76% difference observed when analysts re-rated their own scans after one month. Despite these discrepancies, the variability in measurements was relatively low and primarily attributed to factors like breathing artifacts.

**Conclusion:**

The process of using MRI to derive biomechanical measures, particularly for bony structures, demonstrates robust reliability. Variability in measurements is minimal even under challenging conditions, supporting the use of MRI for biomechanical assessments.

## Introduction

Since the 1980’s, magnetic resonance imaging (MRI) has gained popularity as a diagnostic tool for musculoskeletal disorders [1], particularly to assess patient lumbar spinal health. MRI has benefits for imaging the musculoskeletal system [2–4], facilitating better visualization of anatomic and potentially pathologic structures, including cartilage, bones, and ligaments [2, 5–7]. Improved imaging methods have provided better means to measure low back structures’ size and relative position. Many surgeons rely on MRI as an accurate, noninvasive diagnostic method and rationale for medical decisions, including lumbar disc implants.

However, MRI remains relatively expensive. Considering the role that economics plays in patient management, questions arise regarding when and how often an MRI should be administered and the repeatability and accuracy of the images themselves. Bennet and Miller succinctly describe the importance of this endeavor: “Reliability is the cornerstone of any scientific enterprise. Issues of research validity and significance are relatively meaningless if the results of our experiments are not trustworthy. It is the case that reliability can vary greatly depending on the tools being used and what is being measured. Therefore, it is imperative that any scientific endeavor be aware of the reliability of its measurements.” [8].

Sowell et al. [9] asserted that performing repeated scans on relatively few subjects acquired within the same scan session (i.e., the subject remains in position in the scanner) or within concise scan intervals (e.g., subjects removed from the scanner and then scanned again minutes later) may greatly underestimate the sources of variability within and between studies on MRI-derived measurements. However, such a study, while proposed by Sowel et al., was not conducted. To investigate the reliability and accuracy of MRI and the robustness of the process itself, a comprehensive scan-rescan study was conducted.

Rovaris et al. [10] suggested that scan-rescan variability should be compared with the intra-rater variability with three repeated volume measurements of the same scan. However, evidence of such studies is limited. Existing databases of vertebral and intervertebral dimensions tend to be limited with respect to measures of reliability/repeatability with relatively narrow study populations and/or parameters recorded [11]. In addition, most datasets on comprehensive accurate lumbar vertebrae and muscle geometry have not included sex differences. Both men and women of varying ages were included in several studies [12–14]. The results varied widely among studies, possibly due to the different sample sizes and age groups.

The objective of this study was to assess the inter- and intra-rater reliability of the MRI process itself. Few studies have addressed the overall reliability of MRI measures using distinct scans to measure the same parameters. Our hypothesis was to explore the “worst-case” scenario for biological parameter estimation reliability by comparing separate scans rather than re-examining identical scans a second time.

## Materials and methods

### Subjects

MRI scans of the lumbar intervertebral segments (L2-S1) and trunk/core muscles of thirty-six (36) subjects (20 males and 16 females) were included in this study. All subjects were asymptomatic and older than 19 years of age. All subjects were scanned on a 3 T scanner using a standardized T2 weighted protocol.

None of the subjects reported a history of activity-limiting chronic back or leg injuries, nor experienced any low back pain at the time of the MRI scan. In accordance with Lee et al.’s (1988) exclusion criteria,”Potential participants who had (1) Degenerative changes in the lumbar spine (e.g., crushed vertebral body, trauma, etc.) and/or Erector Spinal muscles (ESMs) (e.g., atrophy); (2) Obvious spinal deformities; or (3) Any known pathology relevant to and likely to alter low back geometry (e.g., scoliosis and tumor) were not included in this study. Subjects were consented in accordance with the Institutional Review Board (IRB) at Auburn University.

### Magnetic resonance imaging

Lumbar MRI scans (L2-S1) of the subjects were performed using a 70 cm Open Bore 3 T scanner (MAGNETOM Verio, Siemens AG, Erlangen, Germany) at the Auburn University MRI Research Center. All subjects were examined in a head-first-supine position (head towards the magnet with hands lying across their abdomen) with arm supports (available upon request) and leg support (mandatory). MRI coil selection included the in-table spine coils and a flexible body coil placed in between the pubic bones at the middle-lower anterior region of the abdomen. The flexible body coil was secured with straps to maximize signal consistency. The imaging protocols included a standard morphological T2-weighted turbo-spin-echo (TSE) sequence in the sagittal plane, Axial Continuous T2-weighted TSE, and Axial Multi-group T2-weighted images with the following parameters:

- T2-weighted spin-echo with a repetition time (TR) of 3440 ms and an echo time (TE) of 41 ms.
- The section thickness was 3 mm with 385 FoV read and 100% FoV phase.

All subject survey and MRI data were anonymized and linked using a unique subject ID unrelated to their personal information. Data were archived and stored on a secure server.

### MRI reading procedure

**Fig. 1:**
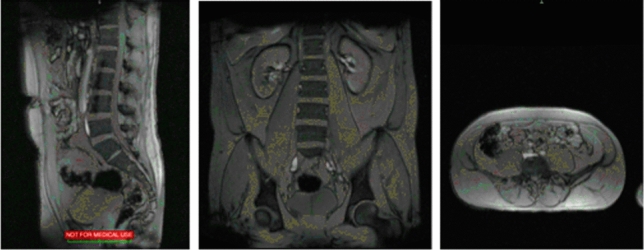
Localizer MRI Scan

Two researchers with experience collecting low back MRI images collected and measured all of the MRI data. One of the operators performed a localizer scan to verify subject placement (aligned straight on the scanner). A localizer scan was obtained to assist in subject placement and allow the analyst to focus on relevant regions of interest (see Fig. 1).

The two MRI analysts reviewed the scans separately. Data were collected from two studies [15, 16] (n = 26 and n = 10, respectively). Images were analyzed a second time after one month. The order of presentation of images was randomized for each analysis. Both analysts were blinded to subject identity. During the MRI interpretation, which considered all MRI sequences, the operators could freely adjust image brightness, contrast, and zoom, to select the slices that they felt were best for measuring the parameters of interest (e.g., disc size, muscle lever arms, etc.).

**Fig. 2:**
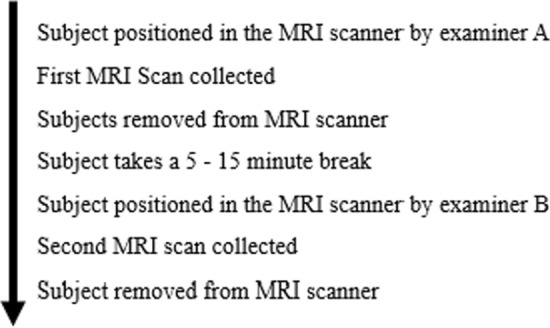
MRI Scan/Rescan Procedure

In order to evaluate MRI reliability, repeated scans with short inter-scan time intervals were performed. Reliability for the entire process was evaluated using a worst-case scenario that compared two distinct scans of the same subject with different analysts positioning subjects and performing each MRI scan (imaging protocol is shown in Fig. 2). In addition, the analysts measured each scan using Osirix^©^ software; their own scans, and the scans performed by their colleague.

### Measurement of lumbar regions (L2-S1)

**Fig. 3:**
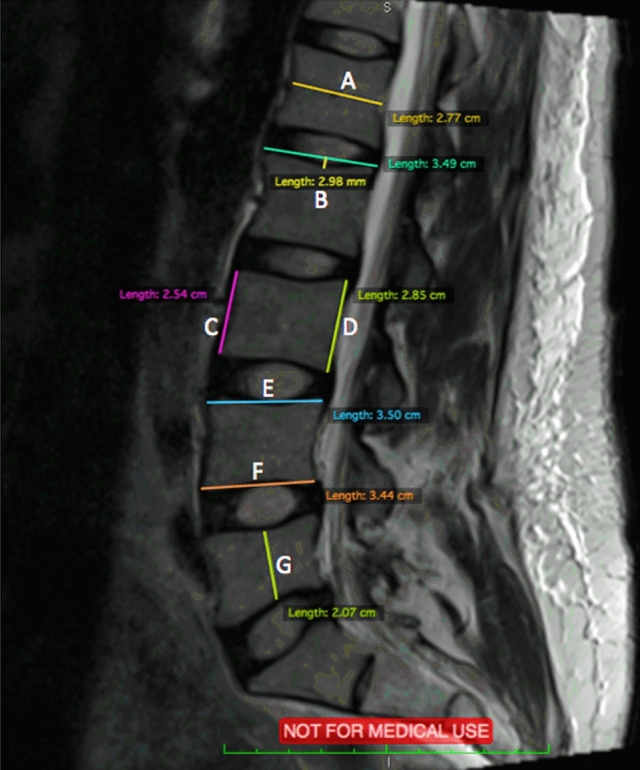
Sagittal MRI scan with measurements of A-F

**Fig. 4:**
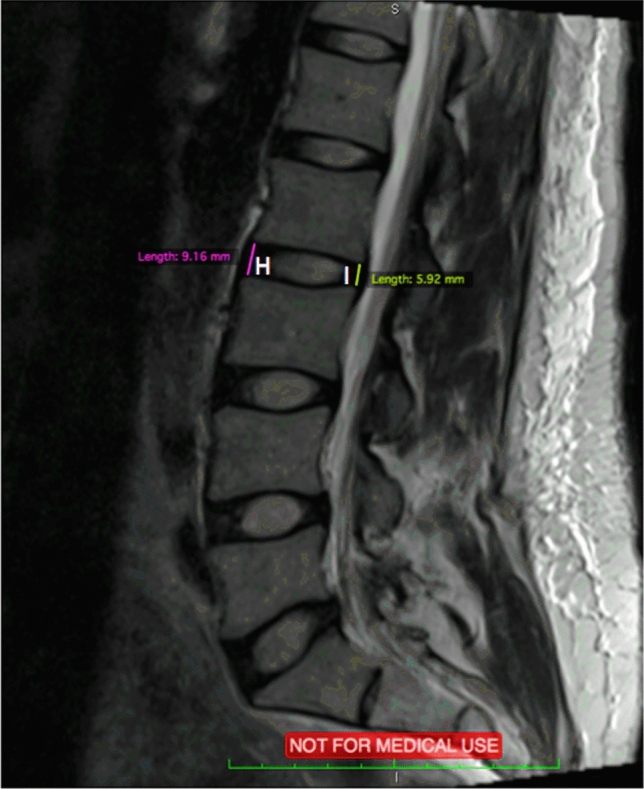
Sagittal MRI scan with measurements of H and I

**Fig. 5:**
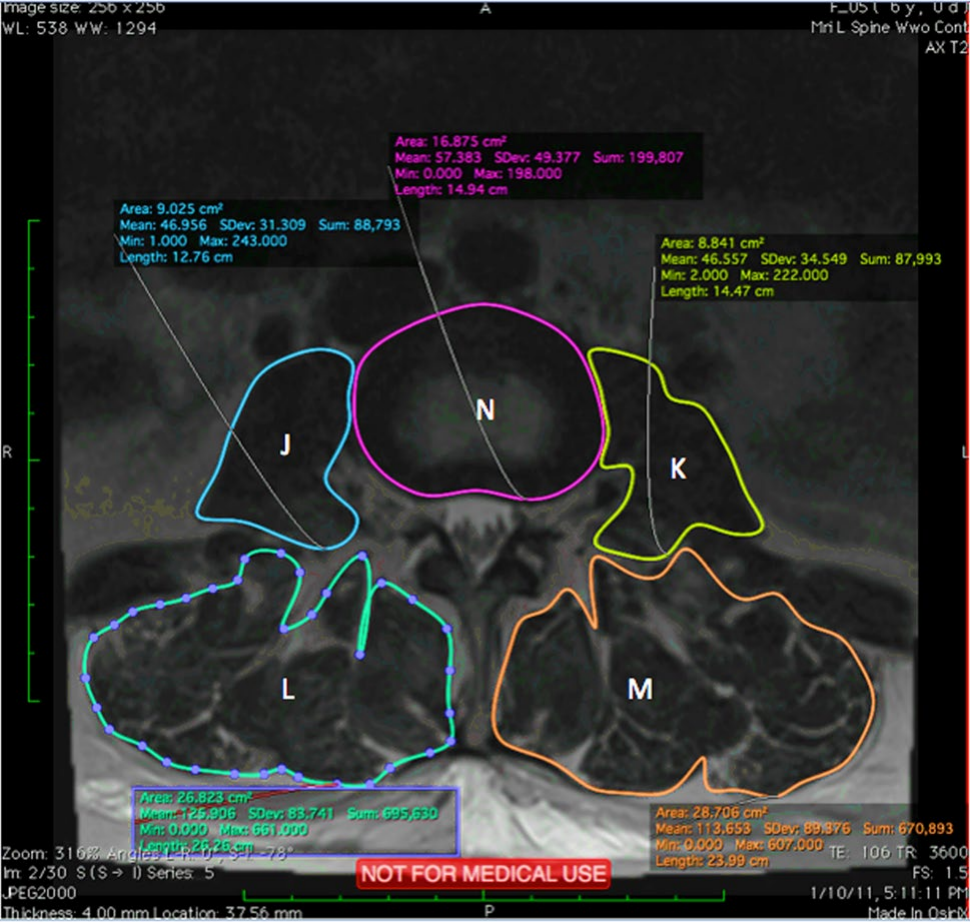
Axial MRI scan measurements of J–N

Axial and Sagittal MRI scans were analyzed using an open-source digital imaging and communications in medicine (DICOM) software, OsiriX, 8.0.1, 32bit, (Bernex, Switzerland). At each spinal level from L2 to S1, 14 measurements were performed and shown in Figs. 3, 4, and 5. Abbreviations are used for these 14 measurements and are shown in Table 1.

**Table 1:** Abbreviations for measurements

Description (Fig. representation)	Abbreviation
(A) Sagittal Vertebrae Body Width	SVBW
(B) Concavity Height	CH
(C) Anterior Vertebrae Height	AVH
(D) Posterior Vertebrae Height	PVH
(E) Superior Vertebrae Body Length	SVBL
(F) Inferior Vertebrae Body Length	IVBL
(G) Sagittal Vertebrae Body Height	SVBH
(H) Anterior IVD Height	AIVDH
(I) Posterior IVD Height	PIVDH
(J) Cross Sectional areas of Psoas Right	PR
(K) Cross Sectional areas of Psoas Left	PL
(L) Cross Sectional areas of Erector Spinae Right	ESR
(M) Cross Sectional areas of Erector Spinae Left	ESL
(N) Disc Size	DAL

### Statistical analysis

The agreement between the examiners (inter-rater) and within each examiner (intra-rater) was analyzed using intra-class correlation coefficients (two-way mixed models: ICC_3,1_) where analysts were fixed variables and subjects were random variables. Muscle Cross-sectional Area (CSA) and Intervertebral Disc (IVD) measurements were stacked together (from L2 to S1 level) to estimate an overall ICC value rather than estimating an individual score for each IVD level. Combining these measurements from multiple levels of the spine (L2 to S1), we obtain a more comprehensive assessment of the overall relationship between muscle CSA and IVD characteristics. In addition to this, aggregating data across multiple levels increases the sample size and statistical power of the analysis. These analyses were performed in SPSS (version 19). Bland–Altman plots were drawn to better visualize the data.

Mean Absolute Percent Differences (MAPDs) were calculated as:

$$MAPD= \frac{\sum_{i=1}^{n}\left|\frac{\left({X}_{i}- {Y}_{i}\right)}{\left({X}_{i}+ {Y}_{i}\right)/2}\right|}{n} \times 100$$

where ${X}_{i}$ is the first measurement, ${Y}_{i}$ is the second measurement, $n$ is the total number of observations.

Since ${X}_{i}$ and ${Y}_{i}$ are two distinct measurements and there is no ground truth, the scores were averaged when estimating error to minimize bias.

## Results

### Descriptive statistics

In total, 144 MRI scans were obtained from 36 subjects. Table 2 presents the demographic data (age, sex, height, and weight). The average age was 23.7 years for males (SD 3.1) and 25.4 years for females (SD 4.8). At each lumbar level (L2-S1), measurements were taken for each IVD and Vertebral body by manually identifying and tracing the actual shape of the structures.

**Table 2:** Mean (SD) anthropometric and demographic data for the male and female subjects

Sex	Age (yr)	Height (cm)	Weight (kg)	BMI (kg/m^2^)
Female (*n* = *16*)	25.4 (4.8)	164.4 (6.6)	64.6 (9.4)	24 (3.9)
Male (*n* = *20*)	23.7 (3.1)	178.2 (8.9)	75.5 (12.2)	23.8 (3.6)

### Scan agreement

Two analysts measured all parameters three times with at least one month between repeated measurements of the same scan to assess the reliability and the repeatability of measurements. Data from six sets of measurements were compared. For each lumbar region analyzed (e.g., L4-L5), there were 50 distinct slices from which researchers could choose to perform their measurements. In order to test the reliability of the overall process, specific image slices were *not* pre-selected for the analysts prior to measurements. Each observer chose the slice they thought most appropriate for measuring the parameters. A comparison of different scans by operators and reviewed/measured by different analysts had not been previously conducted using a substantive sample size. The agreement of analysts in choosing the same slice or within one slice is shown in Table 3. The results show that the same slice was selected 61% of the time, and selections were within one slice over 90% of the time. The agreement was highest when researchers reanalyzed their own scans and was lowest but still excellent when they measured each other’s scans. Analysts waited at least one month between analyses of the same scans, with scans presented in random order each time.

**Table 3:** Probability of selecting identical slice for analysis

Analysis	Absolute agreement	± 1 slice
Within analyst	0.79	0.98
Between analysts	0.50	0.85
Overall	0.61	0.90

Table 3 shows that when analysts repeated an analysis of a particular scan, they selected the same slice 79% of the time. Different analysts reviewing the same scan selected identical slices 50% of the time. Overall, it should be noted that analysts were within one slice of each other’s scans 90% of the time and did not differ by more than two slices (6 mm).

The mean absolute errors for each IVD level were reported as percentages in Tables 4 and 5. The results show that bony structures have lower error percentages except for AIVDH and PIVDH. In general, the smaller structures had bigger percentage errors. Moreover, when the analysts evaluated their own scans, error percentages decreased.

**Table 4:** Summary of Bland–Altman analyses

Variables	Correlation between analysts	Mean error (cm)	Standard Deviation (cm)	% difference	Limits of agreement = Upper LOA—Lower LOA
AVH	0.71	− 0.03	0.20	2	0.07
PVH	0.73	− 0.09	0.19	7	0.18
SVBW	0.79	− 0.10	0.28	7	0.20
CH	0.48	0.03	0.62	3	0.06
SVBL	0.64	− 0.11	0.24	7	0.22
IVBL	0.87	− 0.07	0.31	5	0.13
SVBH	0.67	− 0.03	0.20	3	0.06
AIVDH	0.60	− 0.36	1.67	10	0.72
PIVDH	0.57	− 0.25	0.90	11	0.50
PR	0.69	− 0.30	1.75	5	0.60
PL	0.69	− 0.43	1.75	7	0.87
ESR	0.76	1.99	2.63	22	3.99
ESL	0.77	1.59	2.51	18	3.19
DAL	0.62	− 0.33	0.95	5	0.66

*Anterior Vertebrae Height (AVH); Posterior Vertabrae Height (PVH); Sagittal Vertebrae Body Width (SVBW); Concavity Height (CH); Superior Vertebrae Body Length (SBVL); Inferior Vertebrae Body Length (IVBL); Sagittal Vertebrae Body Height (SVBH); Anterior IVD Height (AIVDH); Posterior IVD Height (PIVDH); Cross sectional areas of Psoas Right (PR); Cross sectional areas of Psoas Left (PL); Cross sectional areas of Erector Spinae Right (ESR); Cross sectional areas of Erector Spinae Left (ESL); Disc Size (DAL)

**Table 5:** Mean Absolute Percentage Difference (MAPD) for Measurements of Intervertebral Discs and Muscles

Dimension	Set of scan slices/Analyst	L2/L3	L3/L4	L4/L5	L5/S1	Overall
AIVDH	Same/Same^1^	5.66	5.40	4.00	3.92	4.74
	Different/Same	7.00	5.95	5.87	5.69	6.13
	Same/Different	13.81	11.42	12.11	13.00	12.58
	Different/Different^2^	13.62	11.30	11.87	13.42	12.55
PIVDH	Same/Same^1^	7.42	6.52	5.61	5.87	6.35
	Different/Same	9.72	6.45	7.14	6.62	7.48
	Same/Different	15.97	14.10	12.39	14.24	14.18
	Different/Different^2^	15.95	13.68	12.36	14.58	14.14
CH	Same/Same^1^	8.12	7.33	6.82	7.09	7.34
	Different/Same	8.61	8.69	7.71	10.31	8.83
	Same/Different	17.06	17.26	16.13	25.99	19.11
	Different/Different^2^	16.06	16.99	15.96	24.99	18.50
Psoas R	Same/Same^1^	7.28	6.76	5.70	6.77	6.63
	Different/Same	12.25	7.96	6.27	6.50	8.24
	Same/Different	17.22	14.08	9.89	8.96	12.54
	Different/Different^2^	17.07	14.71	9.94	8.71	12.61
Psoas L	Same/Same^1^	8.34	6.71	5.86	6.94	6.96
	Different/Same	12.57	8.69	5.96	7.83	8.76
	Same/Different	20.74	14.55	9.27	8.87	13.36
	Different/Different^2^	20.61	14.49	8.82	8.41	13.08
Erector Spinae R	Same/Same^1^	5.00	4.45	6.46	6.97	5.72
	Different/Same	5.87	5.29	8.13	14.74	8.51
	Same/Different	5.56	6.09	13.33	26.98	12.99
	Different/Different^2^	5.92	6.64	13.49	24.51	12.64
Erector Spinae L	Same/Same^1^	4.77	5.39	5.07	8.51	5.94
	Different/Same	6.65	5.97	6.91	12.64	8.04
	Same/Different	6.67	5.26	12.98	21.06	11.49
	Different/Different^2^	6.74	5.70	11.99	18.63	10.76
Disc size	Same/Same^1^	2.41	3.32	2.60	3.68	3.00
	Different/Same	3.73	3.42	3.31	4.31	3.69
	Same/Different	4.22	3.84	4.51	5.58	4.54
	Different/Different^2^	4.98	4.23	4.59	5.36	4.79

^1^Expected “best case” (same set of scan slices analyzed by the same analyst)

^2^Expected “worst case” (different set of scan slices analyzed by different analyst)

The ICCs for intra-rater reliability show high to excellent (0.806–0.989) agreement for both analysts and are reported in Table 6. The first sets of measurements for both analysts were compared to evaluate inter-rater agreement. The ICC results indicated high to excellent agreement for most of the measurements except PIVHD, SVBL, Disc Size, and CH. It should be noted that CH is a two-step measurement requiring analyst judgment [16], therefore can be more subjective then the other part measurements.

**Table 6:** Intra-class Correlation Coefficients Table for Inter- and Intra-rater Reliablity

Dimension	Intra-rater reliability for analyst 1 and analyst 2				Inter-rater reliability	
	Analyst 1		Analyst 2		Analyst 1 and 2	
	ICC3,1	95% CI	ICC3,1	95% CI	ICC3,1	95% CI
SVBW	0.965	(0.953–0.974)	0.964	(0.952–0.973)	0.856	(0.741–0.912)
CH	0.939	(0.916–0.955)	0.849	(0.793–0.890)	0.608	(0.494–0.702)
AVH	0.918	(0.892–0.938)	0.939	(0.919–0.954)	0.845	(0.657–0.917)
PVH	0.946	(0.928–0.959)	0.924	(0.890–0.946)	0.806	(0.396–0.913)
SVBL	0.896	(0.863–0.922)	0.948	(0.931–0.961)	0.732	(0.474–0.847)
IVBL	0.968	(0.957–0.976)	0.984	(0.979–0.988)	0.908	(0.866–0.936)
SVBH	0.958	(0.943–0.968)	0.950	(0.934–0.963)	0.835	(0.768–0.882)
AIVDH	0.964	(0.950–0.974)	0.959	(0.943–0.970)	0.832	(0.686–0.901)
PIVDH	0.821	(0.759–0.868)	0.897	(0.860–0.925)	0.710	(0.594–0.793)
Psoas R	0.944	(0.924–0.960)	0.975	(0.966–0.982)	0.888	(0.809–0.930)
Psoas L	0.963	(0.949–0.974)	0.979	(0.971–0.985)	0.908	(0.846–0.942)
Erector S. R	0.968	(0.955–0.977)	0.989	(0.985–0.992)	0.911	(0.835–0.947)
Erector S. L	0.967	(0.954–0.976)	0.988	(0.983–0.991)	0.925	(0.891–0.948)
Disc Size	0.806	(0.739–0.857)	0.854	(0.802–0.893)	0.733	(0.580–0.824)

### Bland–Altman analysis

**Fig. 6:**
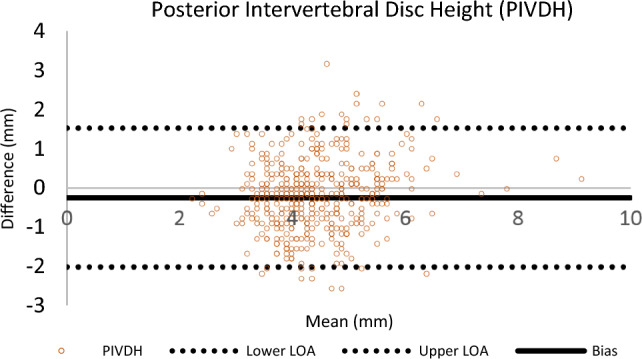
Bland Altman Plot of Posterior Intervertebral Disc Height** (**PIVDH) for Inter-rater Reliablity of Analyst 1 and 2

The Bland–Altman method for repeated measurement was performed to evaluate inter-rater reliability of the analysts for all regions. A representative Bland–Altman plot (Posterior Intervertebral Disc Height [PIVDH]) is shown in Fig. 6. A summary of the Bland–Altman analyses are in Table 7. The main purpose of using these plots is to visualize the agreement between analysts. In these plots, the Y-axis represents the difference between researchers’ readings, and the X-axis represents the average differences between analyst readings. Bias represents the mean of the differences in all measurements. The lower and upper levels of agreement (LOA) represent the mean difference’s 95% confidence interval (± 1.96 SD).

**Table 7:** Mean Absolute Percentage Difference (MAPD) for Measurements of Vertebral Bodies

Dimension	Set of Scan Slices/Analyst	L2	L3	L4	L5	S1	Overall
SVBW	Same/Same^1^	2.31	2.38	2.55	2.76	4.50	2.90
	Different/Same	3.78	2.91	3.06	3.32	6.80	3.98
	Same/Different	4.45	4.72	6.28	6.48	9.60	6.31
	Different/Different^2^	4.89	4.75	5.82	6.62	9.41	6.30
AVH	Same/Same^1^	3.08	2.58	2.68	2.64	2.83	2.76
	Different/Same	3.68	3.10	3.29	3.48	4.21	3.55
	Same/Different	4.23	3.38	3.84	4.10	4.82	4.07
	Different/Different^2^	4.69	3.41	3.65	3.97	4.54	4.05
PVH	Same/Same^1^	2.48	2.50	2.39	2.72	3.23	2.66
	Different/Same	3.31	3.04	2.89	3.48	4.63	3.47
	Same/Different	4.56	4.15	4.58	5.13	6.87	5.06
	Different/Different^2^	4.35	4.04	4.09	4.34	5.85	4.53
SVBL	Same/Same^1^	2.24	2.40	2.05	2.21	3.41	2.46
	Different/Same	3.82	2.71	2.62	3.12	4.51	3.36
	Same/Different	4.73	4.16	5.19	5.91	5.73	5.14
	Different/Different^2^	5.74	4.30	4.44	4.98	5.54	5.00
IVBL	Same/Same^1^	2.05	2.10	2.51	3.06	6.92	3.33
	Different/Same	3.34	2.85	3.16	3.90	7.90	4.23
	Same/Different	4.32	4.86	5.07	7.07	14.25	7.11
	Different/Different^2^	4.47	4.20	4.35	6.45	14.23	6.74
SVBH	Same/Same^1^	2.53	2.39	2.23	2.24	2.79	2.44
	Different/Same	3.42	3.48	2.91	3.22	3.57	3.32
	Same/Different	4.76	4.78	3.53	3.72	5.78	4.51
	Different/Different^2^	4.93	4.67	3.45	3.63	6.08	4.55

^1^Expected “best case” (same set of scan slices analyzed by the same analyst)

^2^Expected “worst case” (different set of scan slices analyzed by different analyst)

The lowest mean differences were observed in the Anterior Vertebrae Height (AVH) and the Sagittal Vertebrae Body Height (SVBH) by − 0.03 cm. The highest mean differences were observed in the muscle groups (Psoas R, Psoas L, ESR, ESL) and the Disc Size. The highest mean differences are − 0.30, − 0.43, 1.99, 1.59, and − 0.33 (cm^2^), respectively (shown in Table 7). A total of 14 measurements were made.

Positive correlations were exhibited between researchers for all measurements (Table 7). Some of these relationships were stronger than others. As complexity of tracing increases, so does the variability. Analysts have closer agreement in vertebral body measurements where the bony landmarks are highly visible. On the other hand, IVD and muscle CSA tracing is much more difficult. These, less well-defined structures were more difficult to measure and were more impacted by subject movement artifacts during scanning.

## Discussion

Morphometry of the trunk muscle CSAs and vertebral bodies have been used for various purposes such as inputs to biomechanical models or to assist in medical diagnoses. To increase trust in MRI data used for these purposes, the procedures and methodologies used to collect that data should be evaluated.

A scan-rescan analysis using repeated scans with short inter-scan time intervals facilitates the assessment of the reliability of the acquired imaging data and increases confidence in the consistency of results [17]. It has been shown that scan results may sometimes show differences when processed with different techniques. Some studies [18] have argued that repeated MR scanning of the same subject, even if using the same analyst and acquisition parameters, does not result in identical representations due to small changes in subject/image orientation, changes in pre-scan parameters, and magnetic field instability. Morey et al. [18] also stated that these differences might lead to appreciable changes in volume estimates for different structures. The accuracy and repeatability of the measurement techniques themselves, however, have not typically been reported in detail. For example, some medical studies focused on the variability of the diagnosis using MRI scans rather than on the reliabilty of the scans themselves. For example, Herzog et al. [19] had a single subject visit 10 different MRI centers for a diagnosis. While they tested the reliability of diagnoses performed by each center’s medical professionals, they did not compare the actual scans themselves. In the present study, the number of subjects was large enough to explore the repeatability of the measurement process itself and to provide accurate information regarding geometric dimensions of both vertebral structures and muscle CSAs.

To our knowledge, AVH and PVH has been studied more often than the other vertebral measurements. In the current study, these parts have shown excellent ICC values for intra-rater (ICC = 0.918, 0.924) and good inter-rater (ICC = 0.845, 0.806) reliability for AVH and PVH, respectively. Hong et al. [20] evaluated AVH to PVH ratio measured by three observers and reported 0.753 for intra-rater and 0.793 for inter-rater agreement. In another study Yao et al. [21] reported excellent reliability coefficients (0.90–0.99) for AVH, PVH, SVBH, AIVDH, and PIVDH. Data from 40 vertebral bodies and 32 intervertebral discs were obtained from 8 cadavers and measured by three observers in their study. Tang [8] reported a higher ICC for intra- (0.990–0.996) and inter-rater (0.971) reliability of disc size. Data from 40 subjecsts were analyzed by two observers with a one-month interval between measurements.

Muscle CSAs showed slightly higher ICCs, however; they also have higher MAPDs. Higher MAPDs may have resulted from breathing artifacts during the MRI process. Valentin et al. [22] reported slightly higher inter-rater agreement for Psoas muscles (0.94, 0.92; left and right side respectively) and for Erector Spinae muscles (0.96, 0.93; left and right side respectively) based on the data from 10 male subjects collected between L1-L5 level. However, the authors did not report any data for intra-rater agreement.

All parameters that were estimated (e.g., cross-sectional areas, vertebral bodies etc.) demonstrated agreement (positive correlations) between analysts. The results indicated that there is always a positive trend (relationship) regardless of observation type (e.g., “best” [same analyst and same scan] case or “worst" [different analysts and different scans] case). Having a different analyst appeared to make a bigger difference than having a different scan, but agreements were good in all conditions. For example, when analyzing the same scan, analysts chose the identical MRI slice (among 50 slices) on which to perform their measurements 61% of the time and within one slice (3 mm) 90% of the time and were never different by more than 2 slices (6 mm). In other studies, the same slice is typically provided to the analysts, however, the present study demonstrates that, when free to choose, analysts will tend to converge on the same slice anyway [23]. This suggests that previous studies utilizing identical slices should only overestimate ICC slightly, further suggesting that MRI is a repeatable means for estimating structure dimensions.

Tracing muscle CSA is more difficult than vertebral bony structures due to less distinct structural boundaries. There were no significant differences between reading one’s scan and reading someone else’s (see Table 3).

Overall, scan/rescan agreement was very high, including “worst-case” scenarios comparing scans read and performed by different analysts. The results suggest that MRI-derived measurements are very consistent and repeatable.
